# Storage management and wastage of reproductive health medicines and associated challenges in west Wollega zone of Ethiopia: a mixed cross-sectional descriptive study

**DOI:** 10.1186/s12913-021-06291-w

**Published:** 2021-04-01

**Authors:** Oliyad Kebede, Gizachew Tilahun, Desalegn Feyissa

**Affiliations:** 1 School of Pharmacy, College of Medicine and Health Sciences, Mizan-Tepi University, Mizan-Aman, Ethiopia; 2 School of Pharmacy, Faculty of Health Sciences, Institute of Health, Jimma University, Jimma, Ethiopia

**Keywords:** Store management, Medicines expiration, Reproductive health

## Abstract

**Background:**

Keeping proper storage conditions at health facilities is vital to reduce pharmaceutical wastage caused by environmental factors. The expiration of medicines at the health facilities could lead to wastage of potentially life-saving drugs and unnecessary expenditure on the disposal of those expired medicines. Therefore, the aim of this study was to assess pharmaceutical stores and wastage of reproductive health medicines due to expiration in the west Wollega zone of Ethiopia.

**Method:**

We conducted a facility-based cross-sectional study from 15th to 31st July 2019 using quantitative and qualitative data from West Wollega Zone of Ethiopia.

**Results:**

Among 23 health facilities assessed, 17 (73.91%) (4(100%) hospitals and 13(68.42%) health centers) fulfilled desirable storage conditions. Hospitals’ stores had equipment and furniture, fulfilled desirable storage conditions, whereas, a significant number of the health centers’ stores did not comply with desirable storage conditions. Challenges of store management identified were poor store infrastructure and shortage of manpower. The total value of reproductive health medicines wasted due to expire in surveyed facilities was 357,920.52 ETB (12,323.81 US dollars) and the Percentage of Stock Wasted due to Expiration was 8.04%. Levonorgestrel 0.75 mg tablet is the highest in the percentage of stock wasted due to expiry. Factors contributing to wastage due to expiration were supply and demand imbalance.

**Conclusion:**

Reproductive health medicines wasted due to expiration is high compared to the government of Ethiopia’s plan for the year 2018/19. This might imply that the monitoring of this plan is poor. Even though hospitals store management is good, there is a weakness in store management in health centers. This could be due to poor attention given to health centers. Therefore, west Wollega zonal health department should appropriately monitor the wastage of Reproductive health medicines and enforce health centers to follow appropriate storage guidelines. Hospitals and health centers should not accept medicines beyond their need to reduce expiry.

## Introduction

Store management is the management of storehouses and stock information, holding and storage of medicines, and the safe custody and protection of stock. Store is a place where we keep stock in the supply chain [1]. Keeping products in store, guarantees satisfying the future customer demand. Therefore, every business stores a quantity of products for future customers’ demand [2]. Store management includes broader activities such as holding; quality control; training of stores staff; and clerical administration of stores operation [2].

Keeping proper storage conditions at health facilities is important to reduce pharmaceutical wastage. Pharmaceutical products stored in pharmacies with good storage facilities maintained their potency [3]. Proper storage of medicines help to ensure that health facilities protect the shelf life of products, that only high-quality medicines are issued, and reduce wastage due to damaged or expired medicines [4]. Thus the regulatory authorities and pharmaceutical organizations should emphasize the importance of preserving good storage conditions in the facilitation of health care [3]. The management of the store should help the flow of supplies from the source to the end-user in the most reliable and economical way without a significant loss of quality, wastage, or theft [5].

The expiration of medicines at the health facilities is a big concern due to its double burden. It leads to wastage of potentially life-saving drugs and causes unnecessary expenditure on the disposal of those expired medicines [6]. Expiration of drugs could also ultimately result in disruption of health services delivery and poor quality of health services thus constraining the attainment of universal health coverage [7]. It was estimated that about 70% of funds spent on medicines could be lost or wasted. However, by improving the basic pharmaceutical management, it is possible to reduce these losses significantly. For example, studies showed that it is possible to reduce medicines lost by expiration by 3%, and medicines lost by improper storage by 4% [8].

Study conducted in Ethiopia, revealed that the medicine wastage is a persistent problem of public health facilities. Poor storage facilities, improper store utilization, inadequate space, stocking expired medicines with usable products, and poor stock rotation, which led to expiry of medicines [9]. Another study also mentioned; guidelines for the storage and disposal of medicines were not available and not followed at lower levels, the available space was not always well utilized or organized, and the practice of first-to-expire-first-out was followed. It also revealed the problem of expired products at all levels, although did not quantify it [10]. The reproductive health (RH) medicines were emphasized because of their nature supply system. The supply chain system for maternal, neonatal, and child health (MNCH) (which are a major constituent of RH) medicines is inconsistent and had not been integrated into the Integrated Pharmaceutical Logistics System (IPLS) [11]. Since IPLS brought significant improvement in pharmaceutical supply chain of Ethiopia [12], being not integrated into IPLS could affect the store management and wastage due to expire. As far as the knowledge of investigators is concerned there is no research conducted on the store management and value of expired medicines in Ethiopia. All the available literatures did not quantify the value of expired reproductive health medicines and factors associated with storage mismanagement and expiration of these medicines. Therefore, the aim of this study was to evaluate pharmaceutical stores and wastage of reproductive health medicines due to expiration at west Wollega zone. It explored the magnitude of expired RH medicines and associated factors. This will be a baseline study for stakeholders and academicians to take corrective action and conduct further studies.

## Methods

### Study area, design, and sampling technique

The study was conducted in public hospitals and health centres of west wollega zone, Oromia regional state, Ethiopia. West Wollega had 19 administrative woredas and its capital, Ghimbi, is located 441 Km to the west of Addis Ababa. The study was conducted from 15th July, 2019 to 31st July, 2019.

Facility based cross sectional descriptive study design was employed. Quantitative result was supported with qualitative result. During data collection period there were five public hospitals and 68 HCs. Actively giving services for more than 1 year during data collection period were considered as inclusion criteria. All hospitals were included in this study, except one that did not fulfill inclusion criteria. HCs were sampled using Logistics Assessment indicator Tool (LIAT) recommendation. Accordingly, 15% of the health centers were included [13]. But this sample size was less than the number of woredas. So, to get representative sample for all woredas, one health center was selected by lottery method from each woreda using lottery method. Therefor 23 public health facilities were assessed.

Source population were all public hospitals and health centers of west Wollega zone, all pharmacy professionals working in those health facilities, all transaction recording tools (Model 19 and list of expired medicines) and all reproductive health medicines. Study populations were all hospitals and selected health centers, selected reproductive health medicines, all store managers in those facilities, selected pharmacy heads, selected PHCU directors, all hospital medical directors, all supply chain coordinators and selected model 22.

### Data collection, data management, and analysis

Structured questionnaires and checklists adapted from Logistics System Assessment tool (LSAT) and Logistics Indicator Assessment Tool (LIAT) prepared by USAID DELIVER Project were used to collect the necessary data [13, 14]. The data collectors took notes on field notes while observing warehouses and expired medicines.

The data were gathered through self-administrated questionnaires from store managers, by observation & physical count of stocks, and review of relevant documents. The stores were assessed by observing stores and filling the checklist, and taking note on the general condition of the store warehouse. We collected the wholesale price of each medicine from invoices. We cleaned the collected data for completeness and consistency. Then, we entered the data into MS Excel 2016 spreadsheet and statistical package for social science (SPSS) version 20 to encode and analyze. Then, count, frequency, percentage, and percentile ranks were computed. The findings were summarized using tables, and figures.

The qualitative data were gathered through in-depth face-to-face interview with key informants and the audio were recorded. The probing and flexible questions were prepared in English and translated into the region’s working language, Afaan Oromo. Then, each key informant was interviewed approximately for 15–20 min in Afaan Oromo. After data collection, the investigator familiarized with the recorded data by listening repeatedly and taking notes. Then, we translated the result into English. The data were coded in Microsoft word table. Data with similar codes were brought together and organized under themes. The report was produced by quoting KIs’ narration under each theme.

### Data quality assurance

Principal investigators gave 2 days trainings for data collectors and strict instructions. The recruited data collectors were pharmacy professionals (druggists and pharmacists). Investigators also supervised the data collectors during data collection. Incomplete and inconsistent questionnaires were taken back to the facility and the data were re-collected.

### Ethical consideration

This study was conducted in accordance with the declaration of Helsinki. Accordingly, the research was approved by the ethical review board of Jimma University, Institute of Health**.** Then, the Review Board gave us permission to obtain letter of permission from each facility and verbal consent from all participants. The permission to conduct the study was then obtained from each facility administrations and verbal consent was obtained from all participants. We excluded the name of participants to keep confidentiality.

### Measurement of variables

The facilities’ pharmaceutical warehouses were evaluated using good storage criteria. These criteria were adopted from Ethiopian Pharmaceutical Supply Agency monitoring and evaluation manual and logistics indicator assessment tool (LIAT) [2, 13].

Percentage of fulfillment of the storage condition for each facility:

$$ \frac{\mathrm{No}.\mathrm{of}"\mathrm{YES}"\mathrm{responses}\ast 100}{\mathrm{Total}\ \mathrm{number}\ \mathrm{of}\ \mathrm{storage}\ \mathrm{conditions}\ \mathrm{considered}(17)} $$

After calculating the percentage of fulfillment of the storage condition, those facilities that fulfilled the criteria more than/equal to 80% were considered as desirable, whereas, those fulfilled less than 80% were considered undesirable [2].

Percentage of facilities that maintain desirable storage conditions:

$$ \frac{\mathrm{total}\ \mathrm{number}\ \mathrm{of}\ \mathrm{facilities}\ \mathrm{that}\ \mathrm{maintain}\ \mathrm{acceptable}\ \mathrm{storage}\ \mathrm{conditions}\left(>80\%\right)}{\mathrm{Total}\ \mathrm{no}.\mathrm{of}\ \mathrm{facilities}\ \mathrm{or}\ \mathrm{warehouses}\ \mathrm{visited}} $$

Fulfillment of each storage condition criteria: $\frac{\mathrm{Number}\ \mathrm{of}"\mathrm{YES}"\mathrm{responses}\ \mathrm{for}\ \mathrm{each}\ \mathrm{storage}\ \mathrm{criteria}}{\mathrm{Total}\ \mathrm{number}\ \mathrm{of}\ \mathrm{storage}\ \mathrm{facilities}\ \mathrm{surveyed}(23).}$

The total quantity and value of wasted medicines due to expire in 2011 Ethiopian fiscal year (July 8, 2018 to July 7, 2019) were evaluated. The medicines expired before July 2018 and accumulated over time were not considered. The beginning stock plus quantity received of the items during this year was evaluated and used as denominator.

Percentage of Stock Wasted due to Expiration

$$ \frac{\mathrm{Unusable}\ \mathrm{stock}\ \mathrm{of}\ \mathrm{an}\ \mathrm{item}\ \mathrm{during}\ \mathrm{a}\ \mathrm{period}\ \mathrm{one}\ \mathrm{year}\ast 100}{\mathrm{Beginning}\ \mathrm{stock}\ \mathrm{plus}\ \mathrm{quantity}\ \mathrm{received}\ \mathrm{of}\ \mathrm{the}\ \mathrm{item}\ \mathrm{during}\ \mathrm{one}\ \mathrm{year}\ \mathrm{period}} $$

## Results

### Background information of the health facilities

#### Presence of basic infrastructures

Four hospitals and nineteen health centers found in west Wollega zone were surveyed, to evaluate warehouse management and reproductive health medicines expiration. Regarding the availability of basic infrastructures, 11(47.83%) of the surveyed health facilities had tarmac, 19(82.61%) had operational electricity, 16(69.57%) had water and 22(95.65%) had operational telephone during the study period (Table 1).

**Table 1:** Presence of basic infrastructures at public health facilities of west Wollega zone, Oromia region, Ethiopia, July 2019

		Hospital(*N* = 4)		Health centers(*N* = 19)		Total(*N* = 23)	
S. No	Presence of Infrastructure	Yes	No	Yes	No	Yes	No
1	Tarmac to the facility	3 (75%)	1 (25%)	8 (42.10%)	11 (57.90%)	11 (47.83%)	12 (52.17%)
2	Operational Electricity	4 (100%)	0 (0%)	15 (78.95%)	4 (21.1%)	19 (82.61%)	4 (17.39%)
3	Water	4 (100%)	0 (0%)	12 (63.16%)	7 (36.84%)	16 (69.57)	7 (30.43%)
4	Operational telephone	4 (100%)	0 (0%)	18 (94.74%)	1 (5.26%)	22 (95.65%)	1 (4.35%)

Seventy-seven health professionals with different educational levels were giving pharmacy services in assessed facilities. From these, 35(45.4%) were pharmacy technicians. This research emphasized on store managers because they were principal personnel responsible for storage of pharmaceuticals. Majority (78.3%) of the store managers had taken formal IPLS training and 10(43.5%) of them were nurses. Nine (39.1%)) of them had 1 to 5 years of working experience (Table 2).

**Table 2:** Socio-demographic Characteristics of professionals working under pharmacy unit in Public health facilities of west Wollega Zone, Oromia region, Ethiopia, July 2019

S. No	Variables		Hospital Frequency (%)	Health Center Frequency (%)	Total Frequency (%)
1	pharmacy unit staffing	Pharmacist	24 (63.2%)	3 (7.7%)	27 (35.1%)
		Druggist	14 (36.8%)	21 (53.8%)	35 (45.4%)
		Nurse	0 (0%)	14 (35.9%)	14 (18.2%)
		Health Officer	0 (0%)	1 (2.6%)	1 (1.3%)
2	Service year (store manager)	< 1 year	1 (25%)	4 (21.1%)	5 (21.7%)
		1–5 year	1 (25%)	8 (42.1%)	9 (39.1%)
		> 5 year	2 (50%)	7 (36.3%)	9 (39.1%)
3	Store managers trained	IPLS	4 (100%)	14 (73.7%)	18 (78.3%)
		SC Overview	2 (50%)	4 (21.1%)	6 (26.1%)
		Never trained	0 (0%)	5 (26.3%)	5((21.7%)

#### Availability equipment and furniture used in store

All hospitals had the basic equipment and furniture used in pharmaceutical store. However, majority of the health centers had no refrigerator, freezer, sufficient shelves, and office table with chairs (Table 3).

**Table 3:** Availability of equipment and furniture in pharmaceutical store at public health facilities of west Wollega zone, Oromia region, Ethiopia, July 2019

S. No	Name of Equipment and furniture	Hospital(*N* = 4)		Health Center(*N* = 19)		Total(*N* = 23)	
		Available	Not available	Available	Not available	Available	Not available
1	Refrigerator	4 (100%)	0 (0%)	6 (31.58%)	13 (68.42%)	10 (43.48%)	13 (56.52%)
2	Freezer	4 (100%)	0 (0%)	6 (31.58%)	13 (68.42%)	10 (43.48%)	13 (56.52%)
3	Wooden pallet	4 (100%)	0 (0%)	9 (47.37%)	10 (52.63%)	13 (56.52%)	10 (43.48%)
4	Sufficient Shelves	4 (100%)	0 (0%)	7 (36.84%)	12 (63.16%)	11 (47.83%)	12 (52.17%)
5	Lockable cabinet	4 (100%)	0 (0%)	15 (78.95%)	4 (21.05%)	19 (82.61%)	4 (17.39%)
6	Ladder	4 (100%)	0 (0%)	7 (36.84%)	12 (63.16%)	11 (47.83)	12 (52.17%)
8	Computer	4 (100%)	0 (0%)	8 (42.11%)	11 (57.89%)	12 (52.17%)	11 (47.83%)
9	Office table with 2 chairs	4 (100%)	0 (0%)	13 (68.42)	6 (31.58)	17 (73.91%)	6 (26.09%)

#### Availability LMIS tools (forms)

Manual LMIS forms like Bin cards, stock cards, receiving and issuing vouchers (model 19 and 22), Report and Resupply Form (RRF), Internal Facility Report and Resupply Forms (IFRRs) were available and being used by all health facilities. All assessed hospitals were using electronic-LMIS during the study period, while only 6(31.58%) of HCs were using electronic-LMIS (Table 4).

**Table 4:** Availability and utilization of LMIS tools in public health facilities of west Wollega Zone, Oromia regional state, Ethiopia, July 2019

LMIS Form	Available			Utilized		
	Hospitals	HCs	Total	Hospitals	HCs	Total
Bin cards	4 (100%)	19 (100%)	23 (100%)	4 (100%)	19 (100%)	23 (100%)
Stock card	4 (100%)	19 (100%)	23 (100%)	4 (100%)	19 (100%)	23 (100%)
e-Recording system	4 (100%)	8 (42.1%)	12 (52.17%)	4 (100%)	6 (31.58%)	10 (43.48%)
Issuing voucher	4 (100%)	19 (100%)	23 (100%)	4 (100%)	19 (100%)	23 (100%)
Receiving voucher	4 (100%)	19 (100%)	23 (100%)	4 (100%)	19 (100%)	23 (100%)
RRF	4 (100%)	19 (100%)	23 (100%)	4 (100%)	19 (100%)	23 (100%)
IFRR	4 (100%)	19 (100%)	23 (100%)	4 (100%)	19 (100%)	23 (100%)
IPLS SOP Manual	4 (100%)	19 (100%)	23 (100%)	4 (100%)	11 (57.89%)	15 (65.22%)

### Storage condition

**Fig. 1:**
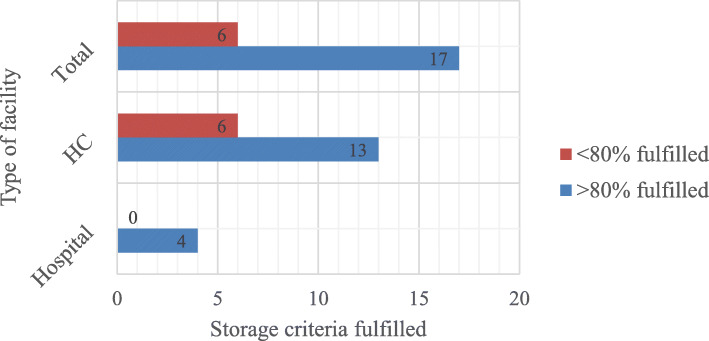
Pharmaceutical stores fulfilling minimum good storage conditions criteria, at public health facilities of West Wollega zone, Oromia Region, Ethiopia, July 2019

Among surveyed facilities, 17 (73.91%) fulfilled desirable storage condition. When classified according to type of facility; all hospitals (100%) and 13(68.42%) health centers fulfilled desirable (> 80%) storage condition criteria (Fig. 1).

The storage condition criteria fulfilled by all the facilities include; stacking products at proper height, store products separately from chemicals and insecticides, protecting products from direct sun light (100%), protecting cartoons from humidity (100%), and securing storage area with lock and key (100%). On the other hand, all the assessed facilities had no functional modern fire safety equipment. However, two (8.70%) of health centers made local fire extinguishers from sand. In majority (52.17%) of the facilities, the storage area is not sufficient (Table 5).

**Table 5:** Number and percentage of warehouses that fulfill desirable storage condition criteria at public health facilities of West Wollega zone, Oromia region, Ethiopia, July, 2019(*N* = 23)

N**o**	Description	Yes	No
**01**	Products that are ready for distribution are arranged so that identification labels and expiry dates and/or manufacturing dates are visible.	18 (78.26%)	5 (21.74%)
**02**	Products are stored and organized in a manner accessible for first-to-expire, first-out (FEFO) counting and general management.	18 (78.26%)	5 (21.74%)
**03**	Cartons and products are in good condition, not crushed due to mishandling. If cartons are open, determine if products are wet or cracked due to heat/radiation (fluorescent lights in the case of condoms, cartons right-side up for Depo-Provera®)	23 (100%)	0 (0%)
**04**	The facility makes it a practice to separate damaged and/or expired products from usable products and removes them from inventory.	18 (78.26%)	5 (21.74%)
**05**	Products are protected from direct sunlight.	23 (100%)	0 (0%)
**06**	Cartons and products are protected from water and humidity.	23 (100%)	0 (0%)
**07**	Storage area is visually free from harmful insects and rodents. (Check the storage area for traces of bats and/or rodents [droppings or insects].)	18 (78.26%)	5 (21.74%)
**08**	Storage area is secured with a lock and key, but is accessible during normal working hours; access is limited to authorized personnel.	23 (100%)	0 (0%)
**09**	Products are stored at the appropriate temperature according to product temperature specifications.	20 (86.96%)	3 (13.04%)
**10**	Roof is maintained in good condition to avoid sunlight and water penetration.	21 (91.30%)	2 (8.70%)
**11**	Storeroom is maintained in good condition (clean, all trash removed, sturdy shelves, organized boxes).	18 (78.26%)	5 (21.74%)
**12**	The current space and organization are sufficient for existing products and reasonable expansion (i.e., receipt of expected product deliveries for foreseeable future).	11 (47.83%)	12 (52.17%)
**13**	Fire safety equipment is available and accessible (any item identified as being used to promote fire safety was considered).	2 (8.70%)	21 (91.30%)
**14**	Products are stored separately from insecticides and chemicals.	23 (100%)	0 (0%)
**15**	Products are stacked at least 10 cm off the floor.	17 (73.91%)	6 (26.09%)
**16**	Products are stacked at least 30 cm away from the walls and other stacks.	20 (86.96%)	3 (13.04%)
**17**	Products are stacked no more than 2.5 m high.	23 (100%)	0 (0%)

### Wastage due to expire and proportion of expired medicines

A total value of reproductive health medicines lost due to expiration in 1 fiscal year in assessed facilities was 357,920.52 ETB (12,323.8 USD). In other words, 78,398 ETB (2699.4USD) in Hospitals and 279,522.52 ETB (9624.4 USD) in assessed HCs were lost due to expire of RH medicines. Whereas, the total value of usable medicines was 4,093,961.2 ETB (140,962 USD). Therefore, the Percentage of Stock Wasted due to Expiration was 8.04%, and ranges from highest wastage rate for Levonorgestrel 0.75 mg tablet (33.08%) to the lowest wastage rate for combined oral contraceptive pills (3.51%) (Table 6).

**Table 6:** Value and percentage of reproductive health (RH) medicines wasted due to expiration from July 8, 2018 to July 7, 2019 at public health facilities of West Wollega Zone, Oromia region, Ethiopia, July 2019

S. No	product Name	Hospital			Health Center			Total		
		Usable	Expired	%age	Usable	Expired	%age	Usable	Expired	%age
1	Male Condom	41,310	0	0	118,357.2	7265.2	5.78	159,667.2	7265.2	4.35
2	Etonorgestrel 68 mg	206,829	5638	2.65	259,516	66,315.6	20.35	466,345	71,953.6	13.37
3	IUCD	70,804.6	12,192	14.69	495,752	31,526	5.98	566,556.6	43,718	7.16
4	Jedelle	55,902.8	405	0.72	46,383	3368	6.77	102,285.8	3773	3.56
5	Norgestrel 0.03 mg	7300.6	2983	29.01	158,837	3565.8	2.20	166,137.6	6548.8	3.79
6	Levonorgestrel 0.75 mg	5315.6	3100.8	36.84	9207.2	4079.6	30.70	14,522.8	7180.4	33.08
7	COC	737,329.8	3192.4	0.43	103,696.6	27,441.2	20.93	841,026.4	30,633.6	3.51
8	Medroxyprogesterone	33,988.2	0	0	128,456.8	12,799.52	9.06	162,445	12,799.52	7.30
9	Oxytocin 10 mg inj.	54,108.8	1639.6	2.94	31,168.4	3405.6	9.85	85,277.2	5045.2	5.59
10	Ringer Lactate	71,932.6	2530	3.40	25,300	3520	12.21	97,232.6	6050	5.86
11	MgSO4	291,072	13,833.2	4.54	91,932.8	6341.4	6.45	383,004.8	20,174.6	5.00
12	Gentamycin	6510.8	240.8	3.57	10,836.2	1568.2	12.64	17,347	1809	9.44
13	Misoprostol	189,552	17,952	8.65	83,424	15,376	15.56	272,976	33,328	10.88
14	Ulcure kit	3314	596.6	15.25	6628	1314	16.54	9942	1910.6	16.12
15	Addis cure kit	11,658.8	0	0	8716.8	1794.2	17.07	20,375.6	1794.2	8.09
16	Addis cure plus kit	19,440	2643.8	11.97	20,995.2	1676.4	7.39	40,435.2	4320.2	9.65
17	Benzanthine Penicillin	2898.6	0	0	15,420	5078.2	24.77	18,318.6	5078.2	21.70
18	Chlorhexidine	4075.8	0	0	267,618	31,208	10.44	271,693.8	31,208	10.30
19	Amoxicillin DT	10,831.6	1792.8	14.20	39,964.6	3133.8	7.27	50,796.2	4926.6	8.84
20	Procaine penicillin	1068	0	0	5340	1204	18.40	6408	1204	15.82
21	ORS	57,112	726	1.262	147,136	30,564.6	17.20	204,248	31,290.6	13.28
22	Zinc sulfate tab	10,163.6	8780.4	46.35	47,101.6	10,693	18.50	57,265.2	19,473.4	25.38
23	TTC eye Ointment	1365.4	151.6	10	5879.2	577.2	8.94	7244.6	728.8	9.14
24	Ceftriaxone	19,260	0	0	9630	1867	16.24	28,890	1867	6.07
25	Vitamin A capsule	6400	0	0	37,120	3840	9.38	43,520	3840	8.11
	**TOTAL**	**1,999,544.6 (68,847.7 USD)**	**78,398 (2699.4USD)**	**3.92**	**2,174,416.6 (748,688 USD)**	**279,522.52 (9624.4 USD)**	**11.39**	**4,093,961.2 (140,962 USD)**	**357,920.52 (12,323.8 USD)**	**8.04**

*NB: Usable = Beginning stock plus quantity received of the item during one-year period*

*1 USD = 29.0430 ETB, Based on ETB to USD Rates on July31, 2019*

Regarding the contribution of each medicine to loss due to expiration, Etonorgestrel 68 mg subdermal (implanon) contributed highest value, holding 71,953.6 ETB/2477.49 USD (20.10%) of expired medicines. Misoprostol with 17,952 ETB/618.12 USD (22.9%), and Etonorgestrel 68 mg subdermal (Implanon) with 66,315.6 ETB/2283.36 USD (23.72%) contributed high value to loss due to expire in assessed hospitals and health centers respectively (Table 7).

**Table 7:** Proportion of expired Reproductive health medicines at public health facilities of West Wollega Zone, Oromia region, Ethiopia, July 2019

S. No	Name of product	Value and Proportion of each expired medicine					
		Hospital(***N*** = 4)		HC(***N*** = 19)		Total(***N*** = 23)	
		Total Value (ETB)	Percentage (%)	Total Value (ETB)	Percentage (%)	Total Value (ETB)	Percentage (%)
1	Etonorgestrel 68 mg	5638	7.19	66,315.6	23.72	71,953.6	20.1
2	IUCD	12,192	15.55	31,526	11.28	43,718	12.21
3	Misoprostol	17,952	22.9	15,376	5.5	33,328	9.31
4	ORS	726	0.93	30,564.6	10.93	31,290.6	8.74
5	Chlorhexidine	0	0	31,208	11.16	31,208	8.72
6	Contraceptive pills	3192.4	4.07	27,441.2	9.82	30,633.6	8.56
7	MgSO4	13,833.2	17.64	6341.4	2.27	20,174.6	5.64
8	Zinc sulfate tab	8780.4	11.2	10,693	3.83	19,473.4	5.44
9	Medroxy-progesterone	0	0	12,799.52	4.58	12,799.52	3.58
10	Male Condom	0	0	7265.2	2.6	7265.2	2.03
11	Levonorgestrel 0.75 mg	3100.8	3.96	4079.6	1.46	7180.4	2.01
12	Norgestrel 0.03 mg	2983	3.8	3565.8	1.28	6548.8	1.83
13	Ringer Lactate	2530	3.22	3520	1.26	6050	1.69
14	Benzanthine Penicillin	0	0	5078.2	1.82	5078.2	1.42
15	Oxytocin 10 mg inj.	1639.6	2.09	3405.6	1.22	5045.2	1.41
16	Amoxicillin DT	1792.8	2.29	3133.8	1.12	4926.6	1.38
17	Addis cure plus kit	2643.8	3.37	1676.4	0.6	4320.2	1.20
18	Vitamin A capsule	0	0	3840	1.37	3840	1.07
19	Jedalle	405	0.51	3368	1.20	3773	1.05
20	Ulcure kit	596.6	0.76	1314	0.47	1910.6	0.53
21	Ceftriaxone	0	0	1867	0.67	1867	0.52
22	Gentamycin	240.8	0.31	1568.2	0.56	1809	0.51
23	Addis cure kit	0	0	1794.2	0.64	1794.2	0.5
24	Procaine penicillin	0	0	1204	0.43	1204	0.34
25	TTC eye Ointment	151.6	0.19	577.2	0.21	728.8	0.20
	**Total**	78,398	**100**	279,522.52	**100**	357,920.52	**100**

### Pharmaceutical store management challenges

The challenges of warehouse mis-management and factors contributing to wastage of reproductive health medicines were identified through in-depth face-to-face interview with relevant key informants and the results were summarized under the following themes.

#### Infrastructure related

Most of the KIs raised infrastructure related problem. Especially, most KIs from the HCs complained that the medical store was a simple room not built for the purpose of medical store. Especially, they explained these factors as a major reason for poor medicines storage. For example, one of the store managers explained the problem as follows:

*“As you see, the store is very congested. The roof is too short and it is difficult to clean daily. The roof needs maintenance. During rainy season, the water penetrates. I have reported to the management and they are discussing. It is long time since I told them. But they complain shortage of budget for maintenance.”*

In addition to the problem of the storeroom, KIs also complained lack of equipment and furniture used in store. Most of KIs from raised that lack of equipment like refrigerator, freezer, and insufficient shelves. This problem is stated by one of the KIs as follows:

*“We are putting cartoons on the ground due to lack of shelves. We have no refrigerator and freezer in our store. Therefore, we store cold chain products in another unit. Since I am not there, I do not know how much the temperature is and they do not record the temperature daily. Since there is frequent electric power interruption, the refrigerator there is not always functional.”*

#### Manpower related

Another challenge identified by most KIs, especially in Health centers was, worker problem. They complained that due to insufficient pharmacy professional at their facility they assigned other professionals to manage pharmaceutical store. For example, one of the KIs expressed it as:

“*We have only one pharmacy technician in our facility. He does all pharmacy duties. We assigned him at dispensary. He also does quantification and procurement in addition to dispensing drugs for patients. So, we assigned midwife nurse at store and the druggist oversees it him.”*

Another KI also stated:

*“I’m nurse. I have not taken any drug management related course during my college study, except pharmacology. However, due to lack of pharmacy professionals in our facility, they assigned me as store manager. Most of the works are strange for me. I am just trying to manage all the works, but sometimes even, I encounter new terms. In addition, I have not taken any training.”*

### Factors contributing to drug expire

#### Supplier related factors

Most of the KIs were complaining the problem of the supplier. They stated that the supplier pushes medicines in bulk without their request.

*“Most of the time PSA and partners dumps a huge amount of family planning medicines with short expire date. We do not need most of these medicines. So, they expire in bulk at our facility.”*

Another KI also explained the problem as:

*“PSA sends near expiry drugs with very essential drug we need, to a driver. If we want to return those near expiry medicines, the driver does not allow us. He obliges us either to receive all products or return all the products. Since some products are very important, we cannot return them; we are obliged to receive those near expiry drugs.”*

#### Demand related

Most KIs raised less customer demand for some family planning methods as contributing factor for expiration. One of the KIs explains this as;

*“Most of our customers prefers medroxyprogesterone (Depo-polivera) over other long-term methods. I think that could be due to its convenience for them. Since the supply from the partners is not need based, they bring other medicines with low demand. Then, those medicines will expire at our facility.”*

## Discussion

Hospitals and health centers store medicines to avoid stockout, to satisfy customer demand, and avoid service interruption. These medicines should be stored in appropriate warehouse, to avoid medicines wastage and theft. While storing medicines, they may expire if not used on time. This research revealed store management and expire of RH medicines. It explored factors that led to store mismanagement like, insufficient storage areas, medical stores not appropriately built for storage of medicines, and manpower challenges. It also revealed factors that led to high wastage rate due to expire: Supply and demand related challenges. Specially, low demand with pushed supply led to high wastage rate for levonorgestrel.

### Availability of equipment and furniture

Basic equipment and furniture are important for properly arranging, putting, and keeping medicines at specified temperature and humidity. It also facilitates first expiry first out (FEFO) principle. The current study revealed that all the hospitals had all basic equipment and furniture used in pharmaceutical store. However, majority of the health centers had no refrigerator, freezer, sufficient shelves, and office table with chairs. These results are better than the study conducted in East Hararge, Ethiopia [15]. The probable reason for difference may be the study setting. The previous study was conducted in woreda stores, while the current is in hospitals and health centers.

Lack of these equipment and furniture had contributed to poor storage management in health centers as, revealed by qualitative result.

### Storage condition

Warehouses used for storage of medicines should fulfill good storage condition criteria to keep the integrity of med and smooth flow of medicines. Among assessed facilities, 17(73.91%) fulfilled desirable storage conditions. This is slightly lower than the study conducted in Nigeria, where all facilities met acceptable storage conditions [16]. The difference might be due to the difference in study setting. The previous study included central medical stores in addition to SDPs. But the current result is better than a similar study conducted by Gurmu and Ibrahim, which reported only 25% of the study facilities fulfilled the criteria of good storage condition (≥80 positive response) [10]. The difference may be because of some criteria like, practicing FEFO, depends on personnel engaged in the store management and were improved in the current study. Almost all the assessed facilities did not have fire safety equipment, even the left two had no modern fire safety equipment rather they made locally from sand, and in majority of the facilities, the storage area was not sufficient.

On the other hand, the storage condition criteria fulfilled by all the facilities include; stacking products at proper height, store products separately from chemicals and insecticides, protecting products from direct sun light, protecting cartoons from humidity, and securing storage area with lock and key. This finding is similar to the study conducted in Nigeria and in East Shewa, Ethiopia [10, 16]. Even though most of the assessed facilities fulfilled desirable storage condition criteria, most of the facilities complain a challenge of poor warehouse infrastructure, and shortage of professionals, as revealed by the qualitative method. Simple room not built for medical store were used as health center warehouse, and even some of them leak during rainy season. This could result in medicines damage, loss of potency, and ultimately ineffective product. This finding deviates from Ethiopian standard operating procedure manual [17]. Again due to lack of trained pharmacy professional, pharmaceutical store is being managed by non-pharmacy professionals, who do not have a training and educational background on pharmaceutical management. However, well-managed pharmaceutical store needs appropriately qualified, trained, and disciplined staffs [1].

### Wastage due to expiration

Reducing wastage of medicines saves the organization money and ensures customers receive quality medicines [18]. In the present study, more than 12 thousand USD were lost in 1 year due to expiration of reproductive health medicines. The result was higher than the results of the study done by Gurmu and Ibrahim in east Shewa, Ethiopia [10]. The difference could be because, the current study was done on program drugs, unlike the previous that was done on key essential medicines, which contains non program drugs. As revealed by qualitative result, program drugs were supplied in bulk without demand of the service delivery points. Again, the logistics system for MNCH commodities (a major portion of RH medicines) is inconsistent and has not been integrated into the Integrated Pharmaceutical Logistics System (IPLS) [11]. Since IPLS brought significant improvements to the supply chain in Ethiopia [12], supply of these medicines was not improved. The overall wastage rate due to expire was 8.04%. The result was higher than Ethiopian health sector plan for 2018/2019 that is 2% [19]. The probable reason for this high wastage rate might be receiving bulk and near expiry medicines, which had low customer demand, as revealed by qualitative method. Similarly, study done in Malawi also identified that the drugs received from donor organizations were based on donor preferences [20]. The current study showed that, Levonorgestrel 0.75 mg tablet contributed highest wastage rate due to expiration. This might be due to its less customer preference as stated by KIs. But supplier push all types of contraceptives irrespective of health centers’ demand.

Since wastage rate in hospitals were lower than that of HCs, it might be due to professionals’ skills assigned in store management. All store managers were pharmacy professionals in hospitals and majority of them took training, while store managers are in HCs were non-pharmacy professionals and did not take training.

### Limitation of the study

The study only included reproductive health medicines and health facilities. Pharmaceutical supply agency, the upper stream of supply chain was not included.

## Conclusion

From this study, we can conclude that Ethiopian government has set plan to reduce expiration of medicines to less than 2% for the year 2018/19, which is by far less than the result revealed by this study. This might imply that the monitoring of this plan at lower-level service delivery points is poor. Again, there is a standard operating procedure and guideline for storage of medicines. Even though, hospitals store management is good, there is a weakness in store management of RH medicines in health centers. This could be due to poor of attention given to the lower-level service delivery points.

Therefore, West Wollega zonal health department should appropriately monitor wastage of RH medicines and enforce health centers to follow appropriate storage guidelines. West Wollega zonal health department should avail equipment and furniture for health centers, and hospitals and health centers should not accept medicines beyond their need to reduce expire.

## Data Availability

The data sets generated and/or analyzed during the present study are available from the corresponding author on reasonable request.

## Abbreviations

FEFO: First expiry first out
HC: health center
IFRR: Internal facility report and resupply form
IPLS: Integrated pharmaceutical logistics system
KI: Key informants
LIAT: Logistics indicator assessment tool
LMIS: Logistics management information system
LSAT: Logistics system assessment tool
MNCH: Maternal, neonatal, and child health
PHCU: Primary health care unit
RH: Reproductive health
RRF: Report and resupply form
SOP: Standard operating procedures
USAID: United States agency for international development
USD: United States dollar
WR: Wastage rate

## References

[1] Management Sciences for Health( MDS-3) (2012). Medical stores management. Managing Access to Medicines and Health Technologies.

[2] EPFSA. The Ethiopian Pharmaceuticals Supply Chain Management System Monitoring and Evaluation Training Manual. 2014.

[3] Shafaat K, Hussain A, Kumar B, Hasan RU, VKY PP (2013). AN OVERVIEW : storage of pharmaceutical products an overview. World J Pharm Pharm Sci.

[4] EPSA. Standard Operating Procedures (SOP) Manual For The Integrated Pharmaceuticals Logistics System In Health Facilities Of Ethiopia. 2015.

[5] Balakrishnan Kokilam M (2015). Ganesh Joshi, Veena Ganesh Kamath H. assessment of pharmaceutical store and inventory Management in Rural Public Health Facilities – a study with reference to Udupi District, Karnataka. Pharm Methods.

[6] Daughton CG (2003). Cradle-to-cradle stewardship of drugs for minimizing their environmental disposition while promoting human health. I. Rational for and avenues toward a green pharmacy. Environ Health Perspect.

[7] Delivering quality health services: a global imperative for universal health coverage. Geneva: World Health Organization, Organisation for Economic Cooperation and Development, and The World Bank; 2018. Licence: CC BY-NC-SA 3.0 IGO.

[8] MSH. Health system in action: Hand book for leaders and managers [Internet]. Management Sciences for Health. 2010.

[9] Gebremariam ET, Gebregeorgise DT, Fenta TG. Factors contributing to medicines wastage in public health facilities of south west Shoa zone , Oromia Regional State , Ethiopia : a qualitative study. 2019;1–7. 10.1186/s40545-019-0192-z PMC6844025 31737281

[10] Gurmu TG, Ibrahim AJ (2017). Inventory management performance of key essential medicines in health facilities of East Shewa Zone , Oromia Regional State, Ethiopia. ukurova Med J.

[11] Woinshet Nigatu, Abebe Bogale, Miraf Tesfaye, Masresha Assefa and FT. Maternal, newborn, and child health logistics system assessment, ethiopia. 2018;(May):1–70.

[12] Shewarega, Abiy, Paul Dowling, Welelaw Necho, Sami Tewfik and YY. Ethiopia: National Survey of the Integrated Pharmaceutical Logistics System. [Internet]. USAID | DELIVER PROJECT, Task Order 4, and Pharmaceuticals Fund and Supply Agency (PFSA). 2015.

[13] USAID (2008). DELIVER PROJECT, Task Order 1. Logistics Indicators Assessment Tool (LIAT).

[14] USAID | DELIVER PROJECT TO 1. Logistics System Assessment Tool (LSAT). Arlington, Va USAID | Deliv Proj Task Order 1\r. 2009;(January).

[15] Gizat Molla Kassie SM (2014). Assessment of pharmaceutical store Management in Woreda Health Offices of west Hararge zone. Int Res J Pharm.

[16] Kolapo: Kolapo, Usman, Elizabeth Bunde ER and EI. Nigeria: Contraceptive Logistics Management System Report. Arlington, Va.: USAID | DELIVER PROJECT, Task Order 1, 2009). Acad Manag J. 2009;(December).

[17] EFHACA. Standard Operating Procedures for Pharmaceuticals Good Distribution and Storage Practices. 2018;

[18] Aronovich D, Tien M, Collins E, Sommarlatte A, Allain L. Measuring supply chain performance: a guide to key performance indicators for public health managers. Arlington: USAID | DELIVER PROJECT, Task Order 1; 2010.

[19] FDRE. Health Sector Transformation Plan 205/16-2019/20.; 2015.

[20] Masefield SC, Msosa A, Grugel J. Challenges to effective governance in a low income healthcare system : a qualitative study of stakeholder perceptions in Malawi 2020;1–16. 10.1186/s12913-020-06002-x PMC7734892 33317520

